# Transactions of the Physico-Medical Society of New York

**Published:** 1821-09-01

**Authors:** 


I. *Transactions of the Physico-Medical Society of New York.* Vol. I. 8vo, pp. 446.

> "Primoque medendi scientia sapientiæ pars habebatur; ut et morborum curatio, et rerum naturæ contemplatio sub iisdem auctoribus nata sit: scilicet iis hanc maxime requirentibus, qui corporum suorum robora quieta cogitatione, nocturnaque vigilia minuerant."
>
> CELSUS.

II. *The Philadelphia Journal of the Medical and Physical Sciences.* Supported by an Association of Physicians, and edited by Dr. N. CHAPMAN. No. 1, for Nov. 1820, 8vo.

THE great utility of medical associations is now universally acknowledged. The discussions which take place among the members themselves are often highly interesting, and tend strongly to excite the minds of all present to the acquisition of knowledge, and to reflection on the topics discussed. The publication of transactions again, when properly conducted, diffuses the benefits of these societies far beyond their original limits; and, through the medium of periodical works, they become the public property of the profession throughout the world.

The Physico-Medical Society of New York is constituted on nearly the same principles as the Medical, and Medico-Chirurgical Societies of London, with the addition of a "committee of abuses." Whether such a committee can ever become operative or useful, in the present frame of society, we have much doubt. That *abuses*, in various senses of the word, exist among the profession in our own country, it would be in vain to deny; yet we hope to see the day when they will be much less common than they are at present. We hope to see the day when *public sentiment* will very effectually check the prominent operations of illiberality, jealousy, dishonesty, disingenuity, and selfishness, which disfigure occasionally a noble and beneficial science —while *general diffusion of knowledge* shall render charlatanism, ignorance, and indolence, such conspicuous objects that they will gradually hide their heads. To accelerate so desirable an event, however, it is necessary that the opposite virtues to the above vices, should not only be inculcated by precept, but exhibited in example.

Periodical literature flourishes on both sides of the Atlantic. Among the numerous candidates for periodical celebrity, a new one burst, or rather *aborted*, into existence, a few months ago, in this metropolis, with the professed view of uniting physic and politics—pathology and plays—metaphysics and farces, "in all the mazes of metaphorical confusion!" The literary bantling was ushered into the world rather before its time, by a favourite protegée of that *discriminating* and *kind-hearted* patron of *osteological* science—the PETRIFIED SKELETON in the British Museum, who, it is said, "grinned horribly a ghastly smile," when a "TREATISE ON RICKETS," superbly done up in a *marble* cover, was laid at his *feet*—we beg pardon, at the ruins of his *metatarsal* bones—by the learned, and very *sensible* author. Whether it was owing to the *cold* reception of DOMINI SKELETON, or the satirical remarks of impertinent reviewers, we know not—but certain it is, that the *dedication* page was violently disrupted from its place, and consigned to the *flames*—where doubtless it received a *warmer* reception. As for the literary abortion, the Medico-Politico-Theatrico-Metaphysical Newspaper that—

"Monstrum horrendum informe cui *lumen ademptum*,"*

it shared the fate of the dedication to the skeleton, in a few days after its birth.

But it seems that it is on American soil only that periodical medicine can come to perfection. "Controlled by hostile feelings (says our cotemporary at Philadelphia) and the *meanest jealousies*, the most enlightened nations of *Europe* perpetually offer proof of a mutual spirit of injustice, in the suppression or depreciation of each others' merits—and more particularly in relation to medical improvements. Too neutral in our position to be warped or influenced by such considerations, *we* are, in this case, the *best* prepared to institute a candid enquiry, and pronounce a just and *impartial* decision." Well, gentlemen, be it so! Far is it from our wish to disturb your happy dreams of optimism, or presume to question the very "*impartial decision*," with which you commence your judicial proceedings. All we can say is this, that if you judge of the British faculty by the sarcastic questions of the northern athenian, which you have adopted as your motto,† you have imitated the French pri-

---

* It is a curious fact that the Medico-Political Newspaper in question, did actually agree with Ulysses's blinded Cyclop, in having but one eye—or rather the socket of an eye, and that in the middle of its forehead. In the dark orbit of the newspaper a snake is seen prowling about, and instead of the intellectual light within, a few glimmering stars are set around it, to render "darkness visible."

† The Philadelphia Journal prefixes the following motto to its first number:—"In the four quarters of the globe, who reads an American soner who, viewing from his PONTON in Portsmouth harbour the *ladies* on Point Beach, drew from thence his picture of female society in England.* In truth, the medical philosophers of this country are not such politicians as often to mix up national prejudices or antipathies with their medical tenets or researches. As far as we ourselves are concerned, we can assure Brother Jonathan that we read his books whenever we can get them, nor have we the slightest wish to rob him of a particle of his reputation. It is true, that we do not put ourselves in a fever about ascertaining the exact period and place when and where an idea was first started, or a fact first discovered. All we want to know is, whether it is or is not a useful idea or fact—and if the former, how best it may be sent diffusively among the profession.

We may here notice the great noise made in this and other countries by some men whose critical researches enable them to discover, from time to time, that many opinions or facts, now considered new, were known in former times. If these men would only look into the book of Nature around them, or the passing stream of existence, they would soon see how inevitable it is that new things should grow old, and old things turn up again as new. This is the lot of every thing on earth—moral and physical. The human mind can embrace, and the human memory retain, but a limited range of knowledge—like the eye, while we travel round this globe, losing one horizon as it gains another. It follows, therefore, that in descending the stream of time, a portion of our knowledge is constantly sinking into oblivion, to make way for new accessions to our stock. If it be objected, that it does not pass entirely out of our reach, since it is chronicled in the records of the age in which it flourished; we answer that, to the great mass of society it is virtually lost, when it requires more time than they can spare for the recovery of it from the dusty annals in which it is buried, amid boundless rubbish. To us indeed, it appears to be a matter of perfect indifference (as

---

book? or goes to an American play? or looks at an American picture or statue? *What does the world yet owe to American physicians or surgeons?*"—Edinburgh Review, No. 65.

* We were lately not a little amused to see an American journalist seriously analyze and highly laud the wretched production of one of the most noted charlatans in this metropolis! We will not wound the feelings of the transatlantic critic, or feed the vanity of the cisatlantic quack, by mentioning the name of either.

far as practical utility is concerned) whether a fact or opinion, *not generally known*, be rescued from the mouldering tomes of antiquity by erudite research, or elicited from the collision of existing circumstances, by plodding industry: We consider both parties as *discoverers*, though one travels *backwards* and the other *forwards* in quest of the prize. In both pursuits the mind is exercised, and that is the main object after all.

I. *Gastrotomy*. In the Philadelphia Journal there is a *lengthy* article (nearly sixty pages of small type) on rupture of the uterus, and the Cæsarian operation, by Dr. Dewees; a gentleman of whom we have more than once made honorable mention in this Journal. We cannot afford space for a regular analysis of this paper, which is principally ratiocinative and historical. Dr. D. combats the opinions of Dr. Denman,* and advocates gastrotomy under certain circumstances, which he endeavours to lay down as guides for our conduct.

Placing no reliance in the powers of Nature to effect a cure, when the child has escaped into the abdominal cavity, he considers that nothing but *prompt* artificial aid can rescue the mother from destruction. The injury which the system sustains by this casualty, is not simply from the lesion of the uterus itself; but also from the additional evils which must follow from inflammation of the peritoneum. To be useful, therefore, we cannot be too early in the removal of the offending bodies from the cavity of the abdomen, since (our author asserts) there never has been a recovery where the fœtus has been allowed to remain. This distressing accident may happen, according to Dr. Dewees, under the following circumstances or modifications.

1st. When the laceration is confined to the body or fundus of the uterus, but penetrates the peritoneum, the child escaping into the abdomen.

2dly. Where the laceration extends only to the peritoneal coat, and the child does not pass into the abdomen. 3dly. Where the laceration is confined to the neck of the uterus and vagina, the child escaping into the belly. 4thly. Where the laceration is confined to the vagina alone, and the child gets into the abdomen. 5thly. Where the 1st and 3d varieties

---

* "When the uterus is ruptured," says Dr. Denman, "at the time of labour, both reason and experience shew, that the patient has a better chance of recovering, by resigning the case to the natural efforts of the constitution, than by any operation or interposition of art."

are complicated with a descent of the intestine. 6thly. Where the laceration may be either in the fundus, body, neck, or vagina, but the child remains either entirely, or in great part, in the cavity of the uterus.

In our efforts to relieve the patient under this afflicting event, we find ourselves restricted to three general modes, viz. either to attempt delivery *per vias naturales*—by gastrotomy, or the Cæsarian operation—or leave the case to the efforts of Nature.

The first mode, though most agreeable to our feelings, is not always proper, or even practicable. Gastrotomy and the Cæsarian section are horrible measures; but when life is at stake, there is strong inducement for the medical attendant to propose, and the patient to submit to desperate attempts to save it. Where this is the only, and anceps remedium, the case should be candidly stated, that no after-blame may attach, and the responsibility should be divided by consultation with our brethren.

Having related several cases from different authorities, of the operation being practised with success, our author goes on to point out the mode to be pursued, under the varied divisions which he has made of ruptured uterus.

In cases similar to 1. of the scale, where the pelvis is well formed, but where the uterus contracts firmly, we should proceed to gastrotomy; for in this case the hand cannot be pressed through the uterus to deliver *per vias naturales.* Where the uterus remains flaccid, however, there may be a possibility of delivering per vias naturales, and this may be attempted. Where the pelvis is deformed, with strong contraction of the uterus, we have no other alternative than gastrotomy.

In cases such as 2, or where the pelvis is well formed, but the uterus contracted, the only chance is gastrotomy, and then cutting through the peritoneal coat of the uterus, so as to free the child from it.

Our author goes on, very systematically, on paper, to lay down rules, which we fear will not be easily remembered or acted on in such trying scenes. But we leave the consideration of this subject to our obstetrical brethren.

II. *Paralysis.* In the same number of the Philadelphia Journal there are some cases related by Dr. Calhoun, illustrative of the use of the tourniquet in palsy. A patient was admitted into the Pensylvania hospital with paralysis of the extensor muscles of the leg, arising from having slept four hours in a sitting posture, with one extremity crossed over the other, and awaking with the usual symptoms produced by pressure on the sciatic nerve. The limb was partially insensible and incapable of motion or of supporting the weight of the body. After two hours these symptoms disappeared; but the extensor muscles of the foot continued paralytic, and the power of motion in them became quite lost.

"The conjecture that the quantity of blood in the paralytic parts was deficient, induced the application of the tourniquet, as an increase of circulation had been observed to follow its removal. The pressure was made for half an hour, so as to stop the pulsation of the artery on the top of the foot, and repeated four times a day. The perspiration of the foot increased during its application, and a glow was felt after its removal. The sensibility gradually returned to the skin over the ancle, (where it before had been lost) the power of motion succeeded, and in about ten weeks the patient was perfectly we l." 132.

Another case occurred to our author, where, in four days after the application of the tourniquet, the disease began to abate. Dr. Calhoun goes on to analyze the effects of pressure by the tourniquet, in a series of experiments of which we shall offer some account to our brethren of the "old world."

The circulation being stopped by means of a tourniquet placed on the upper part of the arm, near the insertion of the deltoid muscle, the temperature fell from 97 to 92 degrees of Fahrenheit—the limb became livid—the power of muscular motion was weakened—sensation gradually became extinct, at the end of twenty minutes, when the experiment was terminated. This experiment was repeated several times, and with nearly similar results. On removing the tourniquet, increased sensibility was evinced by a pricking sensation, a sense of fulness, a sudden glow, and, in most cases, a speedy restoration of the power of motion.

All the powers of the limb, then, being reduced by pressure, increased excitability to the impression of the blood and influence of the brain and nerves is the consequence, as appears from the increased sensation and temperature when the pressure is removed—"thus, by the frequent repetition of the operation, the palsy is cured." Pressure by the tourniquet has been applied to three patients affected with general palsy with good effect; but the remedy appears more peculiarly applicable to local palsy, where internal remedies are objectionable on account of their deranging the functions of the digestive organs.

III. *Hepatic Functions.* We now turn to the transactions of the Physico-Medical Society of New York, but must pass over many articles which, though locally interesting to Americans, are not so much so here. Our readers are pretty well aware that we seldom notice theoretical articles in this Journal; and the reason is, because we can always fill our pages with more useful materials. We shall, in this place, however, glance at a physiological article in the work under review, because there is a very striking coincidence between the opinions of its author and of Dr. J. M'Donnel, in the January number of the Edinburgh Medical and Surgical Journal. The medical world know that the latter gentleman has laboured to shew that a principal use of the liver is to assist the lungs in the decarbonization of the blood. Dr. Pierson has laboured to the same effect, in a paper read to the Physico-Medical Society, on the 5th of March, 1816. We shall introduce an extract to shew the coincidence alluded to.

"The porta collects the blood that returns from the stomach, spleen, omentum, the whole of the intestinal canal, and the mesentery; a surface much greater than that of the whole body.

"This blood has undergone no change at the extreme vessels, like that which takes place on the skin; and is accordingly found to be darker, and to contain much more carbon than the rest of the venous blood.

"The liver is known to separate this carbon, and to discharge it in the composition of bile, of which it is the chief ingredient. Have we not reason, therefore, to believe, that an important use of this viscus is to decarbonize the blood? If it be said, that this doctrine of the function of the liver degrades this organ, from the rank of a secreting, to that of an excreting one, it is answered, that this conclusion does not follow; for it is not denied that in the process of digestion and chylification, and in its operation on the bowels, bile possesses the importance of a peculiar secreted fluid. In what instance, then, is the simple, but salutary economy of nature, more beautifully exemplified than in the process of forming, and the no less important manner of disposing, of the bile!

"I infer, that the liver acts as an assistant to the lungs in decarbonizing the blood, from the absolute necessity of such an assistant.

"The lungs, in the performance of this function, are affected by external circumstances; and are influenced by causes which are extraneous, and over which the system has no controul. These are exercise, and the variations in atmospheric weight and temperature. Now, when we consider how precise the system is, with respect to the quantity of circulating carbon it will contain, without offence; and how liable this quantity is to fluctuate, from unavoidable causes, we should expect to find in the animal economy a contrivance, analogous to what chemists call a valve, or tube of safety; which would permit the escape of surplus carbon, and save the system from fatal injury. Such an office I conceive the liver to perform.

"From what has been said, is it not rendered highly probable that the liver is an organ of more consequence than has generally been supposed; and that, besides the relation in which it stands to the intestines, it also co-operates with the lungs? It is also probable, that, in the fœtal state, the liver supplies, in a measure, the place of the lungs.

"The meconium is, beyond doubt, produced by the liver, and is found in the intestines, during almost the whole period of gestation. By chemical analysis, it is found closely to resemble the adult bile, and to consist chiefly of carbonaceous matter. It is said, that the blood returned from the placenta, by the umbilical vein, is more florid than that in the umbilical arteries. This may be the case, and yet the liver perform the office of lungs; for the business of decarbonization may be, but in part, executed in the lungs, while the liver does the remainder, and perhaps the chief of it. Richerand is of this opinion." 107.

IV. *Efficacy of Emetics in Spasmodic Diseases.* Dr. Joseph M. Smith, in a paper read before the Physico-Medical Society, endeavours—

"*First.*—To show the inefficacy of the common mode of treating the spasmodic symptoms of hysteria and epilepsy.

"*Secondly.*—To exhibit the superiority of *emetics* as antispasmodics.

"*Thirdly.*—To inquire whether their use is not founded on the laws of the animal economy: and

"*Fourthly.*—To notice some of the diseases, in which they may be successfully employed." 130.

Dr. Smith much doubts whether the common antispasmodics, as assafœtida, castor, ether, valerian, opium, &c. mitigate in any degree hysteria—opium perhaps excepted. In plethoric habits, however, this last medicine is often objectionable.

"In plethoric habits, it is customary to bleed before administering opium. This plan is highly judicious. Bleeding will diminish local, as well as general plethora; lessen the impetus of particular determinations, and generally render the system more susceptible of the operation of medicine. Venesection, however, when it is most required, is sometimes either impracticable or extremely inconvenient, on account of the intractable struggles of the patient, during the paroxysms, and the shortness of the intervals. When the operation is performed, and the other means duly employed, it is expected, that the convulsions will soon cease; but how often does it happen, that several hours elapse before they subside?

"The above observations are equally applicable to epilepsy, the clonic spasms of which are similar to those of hysteria, and demand the same treatment, notwithstanding they may arise from very different remote causes. Indeed, the remote causes of both disorders are generally disregarded, so long as the fits succeed each other with rapidity; the first object being to remove the spasms." 133.

The experience which Dr. Smith has had of the utility of emetics in hysteria and epilepsy, enables him to assert, with confidence, that they are more efficacious than any remedy ordinarily employed in convulsions. The rules to be observed in their administration are few and simple.

"If the paroxysms quickly succeed each other, the emetic should be given in divided doses during the intervals. No alteration of the symptoms will take place until the occurrence of nausea; after which, the spasms will be found less violent, and the patient will complain of sickness and languor. As the nausea increases, the spasms become still weaker, and the intermissions longer, and as soon as full vomiting occurs, the paroxysms cease to return. Such is the manner in which the convulsions generally disappear. In some cases, I have seen the fits terminate before vomiting was excited; and in others, noticed several slight ones after the contents of the stomach were ejected; but in no instance have I known vomition to fail in producing the desired effect. In general, between the fits, patients are rational; but it sometimes happens that they are delirious or comatose. When this is the case, there is some difficulty in administering the medicine; but in common, sufficient can be given to answer the intention. Under these circumstances, the first favourable change is a perfect restoration of sense and voluntary motion; the patient expressing great uneasiness about the stomach." 134.

Should the patient be plethoric, or evince much determination to the head, blood should be drawn from the arm, anterior to the exhibition of the emetic, which will render the convulsions more manageable. Should the convulsions manifest a disposition to recur, *post vomitionem,* a dose of laudanum or some antispasmodic will, in general, prevent them.

We shall not follow Dr. Smith through his *rationale* of vomiting in convulsive diseases. If we can ascertain the *quo,* we are not very anxious about the *quo modo,* in the removal of disease, especially where the latter is founded principally on conjecture.

Besides the spasmodic diseases already mentioned, Dr. S. thinks that there are others in which emetics may be advantageously employed, as in spasmodic stricture of the urethra, producing retention of urine, puerperal convulsions, convulsions of children, and tetanus. Of their utility in these diseases, however, our author does not offer any experimental proofs.

The action of vomiting exerts an amazing influence over almost every function of the body, and therefore if opportunely employed, may be made a powerful item in the catalogue of medicinal agents. In respect to vomiting in epilepsy, three instances have come within our knowledge, where the attacks were considerably mitigated, although the emetics were administered in the intervals. We have also seen several instances where the mode and period of epileptic seizures were changed very much by repeated emetics. The suggestion therefore of Dr. Smith ought not to be despised, especially in those cases of epilepsy where a premonition is given the patient, allowing time for the administration of an emetic.

We shall return to the Physico-Medical Transactions in our next number. In the mean time we wish the society every possible success; requesting our brother editors of Philadelphia to erase from their Journal the illnatured and illiberal interrogation of the Northern Critic, as not at all expressing the sentiments of the British faculty. At the same time, brother Jonathan will excuse us if we just hint the impolicy of praising *himself* so immeasureably, as he will thereby leave no room for *others* to perform that friendly office, on proper occasions.

